# Methodological trends in biological assessment of children’s daily stress: A scoping review

**DOI:** 10.1371/journal.pone.0355460

**Published:** 2026-08-10

**Authors:** Sujeong Kang, Eunkyung Kim

**Affiliations:** 1 Department of Human Development & Family Studies, Duksung Women’s University, Seoul, Republic of Korea; 2 Department of Family and Child Welfare, Wonkwang University, Iksan, Republic of Korea; Jawaharlal Institute of Postgraduate Medical Education and Research, INDIA

## Abstract

**Introduction:**

Childhood stress can influence biological stress-response systems and may have long-term implications for development. Although biological measures are increasingly used to assess stress in children, research on biological assessments of daily stress among typically developing children has not yet been comprehensively synthesized across stress types and disciplines.

**Objective:**

This scoping review synthesizes research from the past 20 years on biological assessments of daily stress in typically developing children, distinguishing between chronic and acute stress and integrating evidence across medicine, psychology, and education.

**Methods:**

Following established scoping review frameworks, we searched PubMed, PsycArticles, and EBSCO Education Source for English-language observational and experimental studies from 2005 to 2025. Studies assessing daily stress using biological indicators in typically developing children were included. Review articles, case studies, pilot studies, and intervention studies were excluded. Data were charted on publication year, country, child age, stress type, data collection methods, biological indicators, and assessment context.

**Results:**

Fifty-seven studies met the inclusion criteria. Overall, the number of studies increased between 2005 and 2019, followed by a modest decline between 2020 and 2025. Acute stress studies were more prevalent than chronic stress studies. Chronic stress research most frequently involved preschool-aged children, whereas acute stress studies most frequently targeted upper elementary-aged children. Endocrine measures—particularly cortisol—were most frequently used, followed by autonomic nervous system measures, whereas brain- and immune-related markers remained limited. The use of repeated daily cortisol sampling increased markedly between 2015 and 2019, followed by a modest decline in 2020–2025, while remaining above levels observed in earlier periods.

**Conclusion:**

This review maps methodological trends in biological assessments of daily stress in typically developing children and identifies research gaps across stress types, age groups, biological indicators, and cortisol assessment practices. These findings can inform more comprehensive and methodologically robust approaches to understanding children’s daily stress.

## Introduction

Stress experienced during childhood can leave biological imprints that persist across the lifespan. Stress is conceptualized as a process in which environmental demands are appraised as exceeding an individual’s coping resources, eliciting physiological, emotional, and behavioural responses, consistent with the transactional model of stress and coping [1]. Although stress responses serve important adaptive functions [2], difficulties in regulating or recovering from stress may contribute to adverse developmental and health outcomes with long-term implications.

Several complementary frameworks provide insight into why early stress exposure may have lasting consequences. The Developmental Origins of Health and Disease (DoHaD) and adverse childhood experiences (ACEs) frameworks highlight how early-life environmental conditions and cumulative adversity shape biological systems and developmental pathways [3–5]. Complementing these perspectives, the concept of allostatic load explains the physiological mechanisms through which repeated stress may accumulate as biological wear and tear, ultimately affecting adaptation and health [2]. Together, these frameworks underscore the significance of childhood stress as a key factor associated with later developmental outcomes [6].

Most previous studies on childhood stress have focused on severe stress associated with major life events such as parental divorce, disasters, and abuse. In contrast, comparatively less attention has been given to children’s daily stress experiences [7]. Daily stress refers to frustration-inducing environmental demands encountered in everyday contexts [8]. Although these stressors are generally less severe than major adverse experiences, everyday stressors can still disrupt emotional and physiological regulation [9,10]. Empirical evidence suggests that high levels of family conflict in daily life can disrupt young children’s physiological stress-regulation systems [11], and that elevated daily stress during elementary school predicts increased internalizing and externalizing problems in adolescence [12]. Despite growing evidence that everyday stress experiences have been associated with children’s developmental functioning, research on daily stress in typically developing children remains relatively limited [13].

Accurate assessment is central to advancing research on children’s daily stress. Traditionally, daily stress has been assessed through self-report or parent-report questionnaires. However, concerns have been raised regarding the reliability and validity of such approaches, particularly for younger children whose memory, judgment, and language abilities are still developing. Indeed, prior studies have noted challenges in obtaining reliable self-report data from pre-schoolers and elementary school children [14,15]. Parent proxy reports are also subject to bias, including bias associated with parental mental health or household background characteristics [16]. Moreover, parents tend to underestimate the severity of children’s stress-related problems [17]. Recognizing the limitations, researchers have advocated for multimethod approaches to stress assessment, reflecting a broader shift toward multimethod measurement frameworks in the stress literature [6,18].

Biological indicators have emerged as an important complementary approach for assessing physiological aspects of stress. Stress responses are primarily mediated by two interconnected neurobiological systems: the autonomic nervous system (ANS) and the hypothalamic–pituitary–adrenocortical (HPA) axis [6]. The ANS, particularly its sympathetic branch, mediates rapid “fight-or-flight” responses via catecholamine release, resulting in physiological changes including increased heart rate, blood pressure, and perspiration [19]. In contrast, the HPA axis produces slower but more sustained endocrine responses through cortisol secretion [6]. Reflecting these distinct physiological processes, stress research commonly uses cortisol-based measures to assess HPA activity and autonomic markers such as heart rate, heart rate variability, and skin conductance to assess ANS activity [6].

These biological stress markers vary in assessment methods and interpretation depending on the type of stress, with a primary distinction between chronic and acute stress. Chronic stress refers to stress resulting from conditions that persist over an extended period of time [20,21]. In children, chronic stress originates from both major life events and persistent daily stressors such as peer conflicts, school adjustment problems, or academic demands [20]. Chronic stress is commonly assessed using biomarkers reflecting long-term physiological activity, such as hair cortisol and inflammatory markers [22,23]. In contrast, acute stress refers to short-term stress responses triggered by immediate stressors, ranging from major events to everyday challenges such as public speaking or task failure [20,21]. Acute stress is often examined in standardized laboratory settings or naturalistic stress-inducing situations, and is commonly assessed using rapidly responding biomarkers such as salivary cortisol and sAA [24].

Although chronic and acute stress require different measurement approaches, they are also closely interrelated and should be studied in an integrated manner [6]. Physiological responses to acute stress are generally adaptive mechanisms that prepare the body to cope with threat [19]; however, prolonged exposure to chronic stress may alter the functioning of biological stress-response systems and is linked to atypical physiological responses to acute stressors [25]. Accordingly, biological stress responses should be interpreted with consideration of the dynamic relationship between chronic and acute stress exposure.

Since the early 2000s, studies assessing children’s stress using biological indicators have increased substantially [26,27]. Nevertheless, research mapping trends in biological stress assessment methods remains limited. Existing reviews (e.g., [24,28]) have primarily focused on biomarker guidelines rather than cross-disciplinary research trends and have rarely distinguished between chronic and acute stress. Moreover, most reviews are limited to medical contexts, restricting understanding of typically developing children. Therefore, a comprehensive trend analysis across disciplines is needed.

This scoping review maps research published over the past 20 years that employed biological methods to assess daily stress in typically developing children, with a focus on methodological trends across chronic and acute stress contexts. This synthesis aims to provide an overview of current approaches to biological stress assessment in children, highlighting methodological patterns and research gaps to inform future directions. This study focuses on children aged 3–12 years, a developmental period critical for the establishment of stress-response systems [6]. Infants younger than 3 years and adolescents older than 12 years were excluded due to developmental differences in stress-response systems. These include limited stabilization in early childhood [6,29] and increased pubertal and neurobiological changes during adolescence [30,31], which may reduce the comparability and increase heterogeneity in biological stress markers.

## Methods

### 1. Protocol and registration

This scoping review was conducted following the Preferred Reporting Items for Systematic Reviews and Meta-Analyses extension for Scoping Reviews (PRISMA-ScR) guidelines [32]. The study protocol was registered on OSF [33].

### 2. Eligibility criteria, information sources, and search strategies

This review examined English-language studies published between January 1, 2005, and December 15, 2025, that employed biological methods to measure daily stress in children aged 3–12 years. The original searches were conducted on 16 December 2025 in PubMed, EBSCO Education Source, and PsycArticles to cover biomedical, psychological, developmental and education-related research. The search strategy combined terms to children, stress, and measurement. To enhance comprehensiveness and reduce the risk of missed studies, the search strategy and terms were independently evaluated by two reviewers and one external reviewer with a doctoral degree. The finalized search strategy was applied consistently across databases by one reviewer.

Because the original search strategy excluded studies focused specifically on poverty or low-income populations, supplementary searches incorporating poverty-related terms (e.g., poverty, low-income) were conducted on 20 May 2026. These additional searches were undertaken to ensure that studies involving broader child populations, in which poverty or family income was examined as a variable rather than the primary focus, had not been inadvertently excluded. At this point, one reviewer screened records at the title/abstract level using the same eligibility criteria as the original search. To assess coding reliability, an independent second reviewer screened an overlapping 20% sample of these records, with complete agreement. All potentially eligible full-text reports identified from the supplementary searches were independently assessed by two reviewers, no discrepancies were identified. The full search strategy is in S1 Table.

### 3. Selection of sources of evidence

Screening and selection of evidence sources were conducted in two stages. First, two reviewers developed and agreed upon detailed inclusion and exclusion criteria for study selection (Table 1). A single reviewer screened titles, abstracts, and publication types of records retrieved from the database searches applying these pre-specified and pilot-tested criteria to ensure consistency. Records for which relevance could not be determined based on title and abstract alone were advanced to full-text screening. Duplicates were identified and removed based on title and author information. In the second stage, two reviewers independently assessed the full-text reports for eligibility. Discrepancies were resolved through discussion. Studies were excluded if they met any of the exclusion criteria specified in Table 1.

**Table 1:** Criteria on study selection.

	Inclusion	Exclusion
Population	Children aged 3–12 years	No restriction
Exposure	· Acute stressors (short-term or momentary everyday stressor) · Chronic stressors (long-term or repeatedly experienced everyday stressor)	· Socioeconomic adversity as the primary stressor or sampling criterion (e.g., poverty- or low-income-focused studies) · Health-related conditions or stressors (e.g., physical illness, mental disorder) · Major or traumatic life events (e.g., natural disaster)
Outcome	· Physiological stress reaction (e.g., cortisol, heart rate, blood pressure)	· Behavioral stress reaction only · Perceived stress only
Study design	· Observational studies · Experimental studies	· Review studies · Case studies · Proposal · Pilot studies · Intervention studies

*Note*. Table 1 was modified for the purposes of the present study with reference to the classifications of physiological stress responses reported in review articles by [20,34].

### 4. Data extraction and synthesis

Based on prior studies [34,35], the review aims, and the included studies, we developed a set of categories and their operational definitions to guide data charting (Table 2). Two reviewers developed a standardized Excel-based data extraction form aligned with this coding framework. Using the shared extraction form and coding rules, the reviewers independently extracted and cross-coded data from all included studies. Any discrepancies were resolved through discussion until consensus was reached. Critical appraisal of individual sources was not performed, consistent with scoping review methodology.

**Table 2:** Data charting categories and their definitions.

Category	Subcategory	Definition
Publication periods	2005-2009	Range of 2005–2009
	2010-2014	Range of 2010–2014
	2015-2019	Range of 2015–2019
	2020-2025	Range of 2020–2025
Geographic region	North America	Includes countries in North America (i.e., USA, Canada)
	Europe	Includes countries in Europe (i.e., Germany, Netherlands, Finland, Denmark, Belgium, Sweden, Switzerland, Turkey)
	Asia	Includes countries in Asia (i.e., Taiwan, China)
	Oceania	Includes country in Oceania (i.e., Australia)
Types of daily stress	Chronic stress	Stress experienced repeatedly over an extended period in everyday contexts
	Acute stress	Stress experienced over a short period of in everyday contexts
Age groups	Preschool	Children aged 3–5 years
	Lower elementary	Children aged 6–8 years or in the lower grades of elementary school
	Upper elementary	Children aged 9–12 years or in the upper grades of elementary school
	Mixed-age	Two or three age groups combined (preschool, lower-elementary, and upper-elementary)
	Other	Heterogeneous samples including preschoolers and/or elementary school students alongside adolescents aged 13 years and older; included in descriptive results but not discussed as a distinct child age group because of age heterogeneity
Data collection methods	Biological fluid sampling	Measurement of cortisol, sAA, cytokine, or sIgA
	Physiological sensor attachment	Measurement of cardiovascular, autonomic nervous system, or electrodermal indicator
	Imaging and electrophysiological recording	Measurement of brain activation or brain oscillation
	Complex	Combination of multiple biological measurement methods
Biological indicators of stress	Endocrine	Cortisol or sAA
	Immune	Cytokine or sIgA
	Cardiovascular & Autonomic	Cardiovascular measures (blood pressure, heart rate, pulse rate, and PEP), cardiac Autonomic indicators (HRV, RSA, and CAB), or electrodermal measures (SCLR, and EDA)
	Brain-related	Brain activation or brain oscillation
Stress measurement contexts	Typical day	A day that does not differ in any notable way from participants’ usual daily routines
	Social evaluation	Situations where a participant receives social evaluation for their performance (e.g., speech, math)
	Failure	Situations where a participant faces a task with very high probability of failure
	Tactile	Situations where a participant experiences tactile discomfort (e.g., cold stimuli)
	Complex	Use of multiple stress measurement contexts
	Other	Situations not included in the categories listed above

*Note*. Abbreviations: sAA, salivary alpha-amylase; sIgA, salivary immunoglobulin A; PEP, pre-ejection period; HRV, heart rate variability; RSA, respiratory sinus arrhythmia; CAB, cardiac autonomic balance; SCLR, skin conductance level reactivity; EDA, electrodermal activity.

The extracted data covered key study descriptors and methodological features, including publication periods and geographic region; types of daily stress; age groups; data collection methods; biological indicators of stress; and stress measurement contexts. Geographic regions were classified based on the locations reported in the included studies. The extracted variables were summarized by calculating frequencies and percentages using IBM SPSS Statistics 29. Patterns in biological measures were narratively synthesized over time and across predefined categories.

## Results

**Fig 1:**
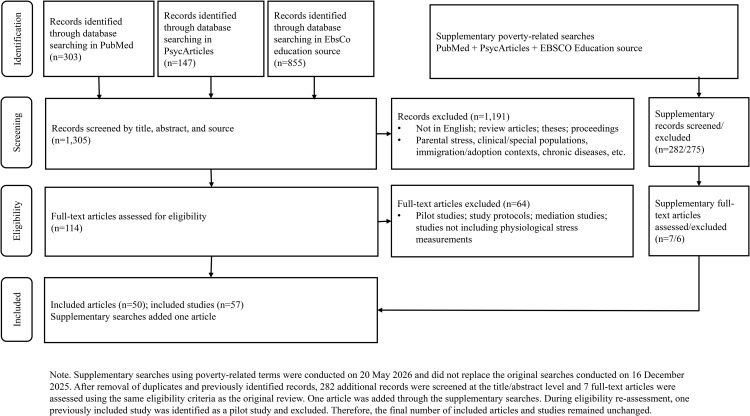
Flow diagram of study selection for the scoping review.

A systematic database search was conducted to identify relevant studies. As shown in Fig 1, the original search yielded 1,305 records, of which 114 reports were assessed for full-text eligibility after title, abstract, and sources screening. Following full-text review, 50 articles, encompassing 57 individual studies, were included in the review. Supplementary searches identified one additional study for inclusion; however, one previously included study was subsequently excluded as a pilot study, resulting in no change to the total number of included studies.

Table 3 presents a summary of the included studies by publication periods, most frequent region, and most frequent stress type (see S2 Table for full study details).

**Table 3:** Overview of included studies by publication period.

Publication periods	Included studies n (%)	Most frequent region (n, %)	Most frequent stress type (s) (n, %)
2005-2009	8 (14.0)	North America (5, 62.5)	Chronic, acute (4, 50.0 each)
2010-2014	17 (29.8)	North America (10, 58.8)	Acute (13, 76.5)
2015-2019	19 (33.3)	North America (17, 89.5)	Acute (16, 84.2)
2020-2025	13 (22.8)	North America (9, 69.2)	Acute (12, 92.3)
Total	57 (100.0)	**–**	**–**

*Note*. Percentages in the “Most frequent region” and “Most frequent stress type(s)” columns were calculated using the number of included studies within each publication period. Full study characteristics, including country-level information, are provided in S2 Table.

### 1. Trends by type of daily stress

The number of studies by stress type over time is presented in Table 4. In total, the number of included studies increased from 8 in 2005–2009 to a peak of 19 in 2015–2019, followed by a decline to 13 in 2020–2025. This pattern was primarily driven by research on acute stress, which increased from 4 in 2005–2009–16 in 2015–2019, before slightly declining to 12 in 2020–2025. In contrast, research on chronic stress remained relatively low and stable, with 4 studies in both 2005–2009 and 2010–2014, followed by a slight decrease to 3 in 2015–2019 and 1 in 2020–2025.

**Table 4:** Trends by type of daily stress n (%).

	2005-2009	2010-2014	2015-2019	2020-2025	Total
Chronic stress	4 (7.0)	4 (7.0)	3 (5.3)	1 (1.8)	12 (21.1)
Acute stress	4 (7.0)	13 (22.8)	16 (28.1)	12 (21.1)	45 (78.9)
Total	8 (14.0)	17 (29.8)	19 (33.3)	13 (22.8)	57 (100.0)

### 2. Trends by participant characteristics

Research trends by participant characteristics were examined with respect to age group and sample size. Table 5 presents the trends by participants’ age group. Mixed-age studies constituted the largest proportion (n = 17, 29.8%), followed by those focusing on preschool children (n = 15, 26.3%), upper elementary school students (n = 11, 19.3%), lower elementary school students (n = 7, 12.3%), and other (n = 7, 12.3%). However, only 4 of the 17 mixed-age studies included preschool children, whereas 15 included lower elementary school children and 16 included upper elementary school children. Consequently, when both age-specific and mixed-age studies were considered, upper elementary school students appeared to be the most frequently represented group (n = 27), followed by lower elementary school students (n = 22) and preschool children (n = 19).

**Table 5:** Trends by participants’ age group n (%).

	2005-2009		2010-2014		2015-2019		2020-2025		Total	
	Total		Total		Total		Total			
	C	A	C	A	C	A	C	A	C	A
P	4 (7.0)		4 (7.0)		3 (5.3)		4 (7.0)		15 (26.3)	
	4 (7.0)	0 (.0)	2 (3.5)	2 (3.5)	1 (1.8)	2 (3.5)	1 (1.8)	3 (5.3)	8 (14.0)	7 (12.3)
L	2 (3.5)		2 (3.5)		2 (3.5)		1 (1.8)		7 (12.3)	
	0 (.0)	2 (3.5)	0 (.0)	2 (3.5)	0 (.0)	2 (3.5)	0 (.0)	1 (1.8)	0 (.0)	7 (12.3)
U	0 (.0)		5 (8.8)		2 (3.6)		4 (7.0)		11 (19.3)	
	0 (.0)	0 (.0)	1 (1.8)	4 (7.0)	1 (1.8)	1 (1.8)	0 (.0)	4 (7.0)	2 (3.5)	9 (15.8)
M	2 (3.5)		6 (10.5)		6 (10.5)		3 (5.3)		17 (29.8)	
	0 (.0)	2 (3.5)	1 (1.8)	5 (8.8)	1 (1.8)	5 (8.8)	0 (.0)	3 (5.3)	2 (3.5)	15 (26.3)
O	0 (.0)		0 (.0)		6 (10.5)		1 (1.8)		7 (12.3)	
	0 (.0)	0 (.0)	0 (.0)	0 (.0)	0 (.0)	6 (10.5)	0 (.0)	1 (1.8)	0 (.0)	7 (12.3)
Total	8 (14.0)		17 (29.8)		19 (33.3)		13 (22.8)		57 (100.0)	
	4 (7.0)	4 (7.0)	4 (7.0)	13 (22.8)	3 (5.3)	16 (28.1)	1 (1.8)	12 (24.6)	12 (21.1)	45 (78.9)

*Note*. Abbreviations: P, preschool; L, lower elementary; U, upper elementary; M, mixed-age; O, other; C, chronic stress; A, acute stress; among the 17 mixed-age studies, 4 included preschoolers, 15 included lower elementary school children, and 16 included upper elementary school children.

When the type of stress was examined by age group (Table 5), studies involving preschool children were almost evenly distributed between chronic stress and acute stress (8 chronic vs. 7 acute). In contrast, acute stress was more frequently examined than chronic stress in all other age groups, including lower elementary school students (0 chronic vs. 7 acute), upper elementary school students (2 chronic vs. 9 acute), mixed-age samples (2 chronic vs. 15 acute), and other age groups (0 chronic vs. 7 acute).

Descriptive tabulation by time period (Table 5) indicates a clear shift in stress type over time. Between 2005 and 2009, studies were evenly split between chronic and acute stress (4 chronic vs. 4 acute). From 2010 to 2014, acute stress studies became more prevalent than chronic stress studies (4 chronic vs. 13 acute), and this pattern persisted in 2015–2019 (3 chronic vs. 16 acute) and in 2020–2025 (1 chronic vs. 12 acute).

Sample sizes varied widely across the included studies. Studies addressing the biological measurement of chronic stress included between 4 and 2,078 participants (median = 94). Most acute stress studies were conducted in laboratory settings, with sample sizes per experimental condition ranging from 14 to 100 participants (median = 53).

### 3. Trends by biological measurement methods

#### 3.1. Trends by data collection methods.

Table 6 presents research trends over time by data collection methods. Overall, biological fluid sampling was the most commonly used approach (n = 29, 50.9%), followed by physiological sensor attachment (n = 18, 31.6%) and complex methods combining more than one data collection approach (n = 10, 17.5%). In terms of temporal patterns, biological fluid sampling was generally more frequently used than physiological sensor attachment, although differences between methods varied across periods and were reversed in 2010–2014. Complex methods appeared in 2010–2014 (n = 1), and were subsequently reported in 2015–2019 (n = 5) and in 2020–2025 (n = 4).

**Table 6:** Trends by data collection methods n (%).

	2005-2009		2010-2014		2015-2019		2020-2025		Total	
	Total		Total		Total		Total			
	C	A	C	A	C	A	C	A	C	A
Biological fluid sampling	5 (8.8)		7 (12.3)		12 (21.1)		5 (8.8)		29 (50.9)	
	4 (7.0)	1 (1.8)	2 (3.5)	5 (8.8)	1 (1.8)	11 (19.3)	1 (1.8)	4 (7.0)	8 (14.0)	21 (36.8)
Physiological sensor attachment	3 (5.3)		9 (15.8)		2 (3.5)		4 (7.0)		18 (31.6)	
	0 (.0)	3 (5.3)	2 (3.5)	7 (12.3)	1 (1.8)	1 (1.8)	0 (.0)	4 (7.0)	3 (5.3)	15 (26.3)
Complex	0 (.0)		1 (1.8)		5 (8.8)		4 (7.0)		10 (17.5)	
	0 (.0)	0 (.0)	0 (.0)	1 (1.8)	1 (1.8)	4 (7.0)	0 (.0)	4 (7.0)	1 (1.8)	9 (15.8)
Total	8 (14.0)		17 (29.8)		19 (33.3)		13 (22.8)		57 (100.0)	
	4 (7.0)	4 (7.0)	4 (7.0)	13 (22.8)	3 (5.3)	16 (28.1)	1 (1.8)	12 (21.1)	12 (21.1)	45 (78.9)

*Note*. Although one study in 2019 employed the “imaging and electrophysiological recording” category, as described in Table 3, it was classified as a complex study because cortisol analysis was also conducted. As no studies used this category on its own, it was omitted from Table 6. Abbreviations: C, chronic stress; A, acute stress.

#### 3.2. Trends by biological indicators.

**Fig 2:**
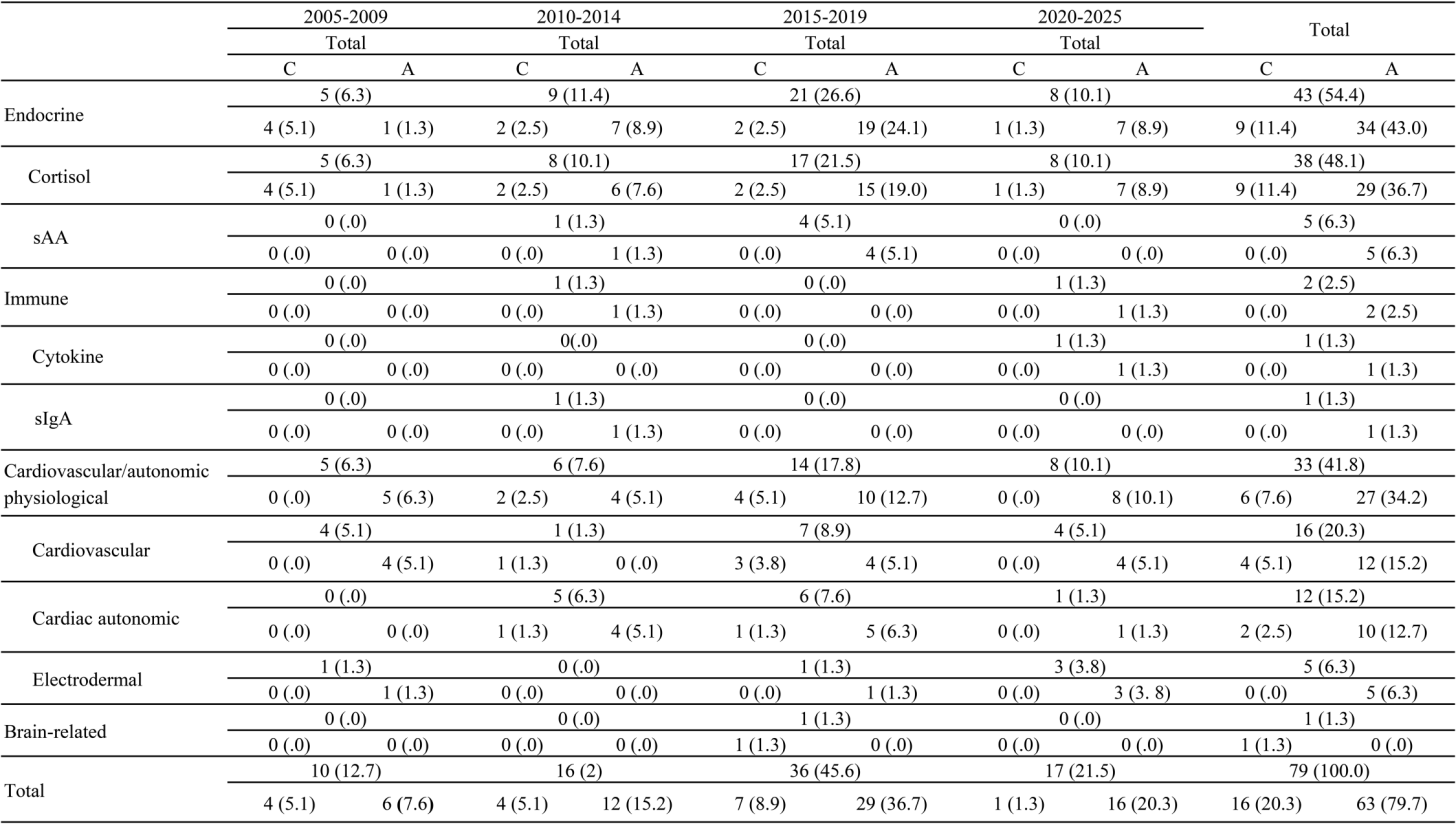
Trends by biological indicators. All values are presented as n (%). Because some studies employed multiple indicators, the total was 79, not 57. Cardiovascular measures include blood pressure (BP), heart rate (HR), pulse rate (PR), and pre-ejection period (PEP). Cardiac autonomic measures include heart rate variability (HRV), respiratory sinus arrhythmia (RSA), and cardiac autonomic balance (CAB). Electrodermal measures include skin conductance level reactivity (SCLR) and electrodermal activity (EDA). Abbreviations: C, chronic stress; A, Acute stress.

Regarding the biological indicators used to assess children’s daily stress (Fig 2), endocrine measures (n = 43, 54.4%) and cardiovascular/autonomic physiological indicators (n = 33, 41.8%) accounted for most assessments, whereas immune (n = 2, 2.5%) and brain-related indicators (n = 1, 1.3%) were rarely reported.

An examination by time period (Fig 2) shows that the two most frequently used indicators—endocrine and cardiovascular/autonomic physiological measures—exhibited an increase from 2005 to 2019, followed by a decline in 2020–2025 (endocrine: n = 5, 9, 21, 8 across four time periods; cardiovascular/autonomic: n = 5, 6, 14, 8 across four time periods). This pattern appears to be consistent with the overall trend observed in the total number of studies presented in Table 4. Studies measuring immune indicators were not reported during 2005–2009. Two studies were identified thereafter, one in 2010–2014 and one in 2020–2025. Brain-related indicators were reported in only one study during 2015–2019.

As shown in Fig 2, among the subcategories of biological stress indicators, cortisol was clearly the most frequently selected indicator (n = 38, 48.1%). Given its widespread use, trends in daily cortisol sampling frequency for stress assessment were further examined (Table 7). The number of cortisol sampling varied widely, ranging from a single measurement [36] to 17 measurements [37]. Because single cortisol measurements can lead to highly unreliable inferences about alterations in the HPA axis, previous studies have generally conducted at least two cortisol measurements [38]. Therefore studies were categorized by the number of cortisol samples collected: 1 sample, 2 samples, and 3 samples or more as shown in Table 7. Results showed that studies employing one or two cortisol samples were generally infrequent across all time periods (one sample: n = 0–1 per period; two samples: n = 1–3 per period). However, a notable increase in studies employing three or more cortisol measurements was observed in the 2015–2019 period (n = 4, 4, 15, 7 across four time periods). Among studies that measured cortisol three or more times, the maximum number of assessments was 17, with a mean of 5.96 measurements.

**Table 7:** Trends by the number of daily cortisol sampling n (%).

	2005-2009		2010-2014		2015-2019		2020-2025		Total	
	Total		Total		Total		Total			
	C	A	C	A	C	A	C	A	C	A
1	0 (.0)		1 (2.6)		0 (.0)		0 (.0)		1 (2.6)	
	0 (.0)	0 (.0)	0 (.0)	1 (2.6)	0 (.0)	0 (.0)	0 (.0)	0 (.0)	0 (.0)	1 (2.6)
2	1 (2.6)		3 (7.9)		2 (5.3)		1 (2.6)		7 (18.4)	
	1 (2.6)	0 (.0)	0 (.0)	3 (7.9)	1 (2.6)	1 (2.6)	0 (.0)	1 (2.6)	2 (5.3)	5 (13.2)
3 or more	4 (10.5)		4 (10.5)		15 (39.5)		7 (18.4)		30 (78.9)	
	3 (7.9)	1 (2.6)	2 (5.3)	2 (5.3)	1 (2.6)	14 (36.8)	1 (2.6)	6 (15.8)	7 (18.9)	23 (60.5)
Total	5 (13.2)		8 (21.1)		17 (44.7)		8 (21.1)		38 (100.0)	
	4 (10.5)	1 (2.6)	2 (5.3)	6 (15.8)	2 (5.3)	15 (39.5)	1 (2.6)	7 (18.4)	9 (23.7)	29 (76.3)

*Note*. Abbreviations: C, chronic stress; A, acute stress.

### 3.3. Trends by stress measurement context

#### 3.3.1. Chronic stress.

For chronic stress, measurements were generally conducted on a typical day without any unusual events. Some studies using cardiovascular/ autonomic physiological indicators, which are sensitive to situational influences, assessed children under resting conditions (e.g., [39,40]).

#### 3.3.2. Acute stress.

Table 8 shows trends by acute stress measurement context. Studies employing two or more stress-induced tasks were classified as complex, whereas tasks that did not fit any predefined category were classified as other. As shown in Table 8, the most frequently used measurement context was social evaluation (n = 16, 35.6%), followed by complex tasks (n = 12, 26.7%), other conditions (n = 8, 17.8%), failure conditions (n = 6, 13.3%), and tactile tasks (n = 3, 6.7%).

**Table 8:** Trends by acute stress measurement context n (%).

	Social evaluation	Failure	Tactile	Complex	Other	Total
Preschool	0 (.0)	2 (4.4)	1 (2.2)	2 (4.4)	2 (4.4)	7 (15.6)
Lower elementary	1 (2.2)	2 (4.4)	2 (4.4)	2 (4.4)	0 (.0)	7 (15.6)
Upper elementary	6 (13.3)	1 (2.2)	0 (.0)	0 (.0)	2 (4.4)	9 (20.0)
Mixed-age	6 (13.3)	1 (2.2)	0 (.0)	4 (8.9)	4 (8.9)	15 (33.3)
Other	3 (6.7)	0 (.0)	0 (.0)	4 (8.9)	0 (.0)	7 (15.6)
Total	16 (35.6)	6 (13.3)	3 (6.7)	12 (26.7)	8 (17.8)	45 (100.0)

*Note*. The “other” category included the following stress tasks by age group: in mixed-age samples, media designed to elicit negative emotions (e.g., sadness, fear; [41,42]), dental treatment-related situations [43], and physical exercise [36]; in upper elementary-aged samples, physical exercise [44] and emotion suppression tasks [45]; and in preschool-aged samples, executive function [46,47] and effortful control tasks [47].

When examined by age group, social evaluation contexts were most frequently used in studies involving upper elementary school students and mixed-age samples (each n = 6, 13.3%), followed by studies categorized as other age groups (n = 3, 6.7%). No studies using social evaluation contexts targeted preschool children, and only one study (2.2%) targeted lower elementary school students. This pattern suggests that social evaluation paradigms were used more often in studies involving older children. Failure and tactile contexts were infrequently reported and showed no clear age-related patterns. Complex paradigms and tasks classified in the other category were observed across multiple age groups.

## Discussion

The present scoping review mapped methodological trends in the use of biological measures to assess daily stress among typically developing preschool and elementary school children over the past two decades. It identified patterns in the study of chronic and acute stress and highlighted important gaps in the literature.

With respect to publication trends across all included studies, publication counts generally increased between 2005 and 2019, although a modest decline was observed during the 2020–2025 period. Overall, these findings suggest an increasing use of biological measures to assess stress in children over the past two decades. Several factors may be relevant when interpreting the decline observed in the most recent period. One possible explanation is the COVID-19 pandemic context, during which laboratory-dependent research activities were substantially disrupted [48,49]. Given that many studies assessing children’s stress through biological measures require in-person assessments, physiological measurements, or biological sample collection, research in this area may also have been affected by these disruptions. However, because the present review did not directly examine the causes of publication trends, no firm conclusions can be drawn regarding the factors underlying this pattern.

Next, the review revealed that studies of acute stress have been conducted more frequently than those of chronic stress. This discrepancy may be partly related to the present study’s focus on daily stress in typically developing children. Chronic stress research more often involves high-risk or clinical samples [6], in which prolonged and cumulative stress exposures are more readily observed among children exposed to severe adversities [6,50]. In contrast, studies involving typically developing children appear to be more commonly represented in the acute stress literature.

In addition, methodological considerations may also be relevant to the imbalance between chronic and acute stress research. Assessing chronic stress typically requires indicators that capture accumulated stress over extended periods and careful management of potential confounding factors [51], which can be especially challenging when working with young children. In contrast, acute stress is generally assessed using standardized laboratory-based tasks, making it easier to measure. A representative experimental stress-induction task is the Trier Social Stress Test (TSST), a social-evaluative stress induction paradigm. In the late 1990s, the TSST was adapted for use with children as the TSST-C [52]. Since the 2000s, accumulating evidence supporting the TSST-C’s ability to reliably elicit acute stress and associated biological responses may have contributed to its widespread use in research on acute stress among children.

Despite the predominance of acute stress research, emerging evidence suggests that everyday experiences, such as family interactions, residential environments, and school or childcare settings, may serve as chronic sources of biological stress activation in children [39,40,53]. Theoretical and empirical work on allostatic load suggests that even relatively moderate but repeated stress exposures may contribute to cumulative wear and tear on physiological systems [6] and produce long-term effects by altering biological functioning [11,12]. These findings underscore the importance of expanding research beyond acute stress paradigms to better understand the biological consequences of chronic daily stress in typically developing children.

When examining studies by age of participants, research on chronic stress was more frequently conducted in preschool-aged children. This pattern may be associated with the growing attention to early childhood education and care (ECEC) settings, such as day-care centers and kindergartens, where young children send a substantial proportion of their daily lives. Among the eight chronic stress studies involving preschool-aged children included in this review, five investigated cortisol levels in relation to ECEC attendance (e.g., [53,54]). This research trend may reflect broader contextual factors, including the expansion of ECEC systems in many OECD countries since the 1990s, which has been accompanied by changes in maternal labor force participation and early childhood education policies [55–57]. However, it should be noted that these interpretations are tentative, and other contextual and methodological factors may also underlie the observed research distribution.

In contrast to chronic stress research, where preschool-aged children were more frequently represented than other age groups, acute stress studies were more commonly conducted among upper elementary-aged children. This pattern may be related to the widespread use of the TSST-C among older children and the more limited availability of well-established acute stress paradigms for younger children [58]. The TSST-C involves public speaking and math tasks conducted under observation, in which being evaluated by others acts as the main stressor. Older children are more likely to perceive these situations as stressful, whereas younger children are unlikely to perceive them as threatening [58]. Among the limited number of studies involving younger elementary school-aged children and preschoolers, a variety of stress induction tasks were used, including failure tasks and tactile paradigms. However, no single task could be identified as a representative paradigm for these age groups. Although some studies have proposed experimental paradigms designed to reliably induce acute stress in preschool-aged children [38,59], subsequent validation of these tasks and adoption by other researchers remain limited. Future research should prioritize the development and standardization of age-appropriate acute stress paradigms for preschool and lower elementary school-aged children.

The frequency of biological stress marker use revealed that endocrine indicators accounted for the largest proportion in both chronic and acute stress research, largely due to the predominant use of cortisol. Cortisol is the most widely utilized biomarker in stress research because it can be measured noninvasively through saliva, hair, or urine samples, making it well suited for children and repeated measurements [27]. In addition, extensive reference data and well-established diurnal patterns support reliable measurement and comparative analyses [60]. However, cortisol primarily reflects HPA axis activity and may not fully capture the multidimensional nature of stress responses, which involve interactions among neuroendocrine, autonomic, neural, and immune systems [61].

The second most frequently utilized indicators were cardiovascular/autonomic physiological measures, including cardiovascular, cardiac autonomic, and electrodermal measures. While cortisol reflects HPA axis responses to stress, cardiovascular/autonomic physiological measures reflect ANS responses, thus providing complementary information when measured alongside cortisol [62]. Moreover, they can be recorded noninvasively using sensors, enabling real-time assessment of physiological responses [63]. Taken together, cardiovascular/autonomic physiological measures serve as important complementary measures in stress research particularly for assessing immediate and dynamic physiological responses.

Since the 2010s, a limited number of studies have examined brain-related measures (e.g., [64]) and immune or inflammatory responses (e.g., [65,66]) as biological indicators of daily stress in children. These studies may reflect an emerging effort to broaden the range of biological indicators used in child stress research. Neural measures can provide insight into attentional, emotional, and regulatory processes associated with stress responses, whereas immune and inflammatory markers are considered to capture physiological consequences of chronic stress exposure that are not fully explained by endocrine indicators alone [67]. However, immune and inflammatory markers are primarily collected through blood sampling [24], which limits their feasibility for assessing daily stress in typically developing children. Similarly, brain-related measures remain constrained by limited access to specialized equipment [68]. These constraints highlight the need to improve the accessibility and feasibility of neural and immune-inflammatory assessment methods to support more comprehensive, multidimensional approaches to understanding children’s stress responses.

Finally, trends in daily cortisol assessment frequency indicate greater use of repeated sampling strategies in more recent studies to improve measurement precision. In particular, studies conducting three or more measurements per day increased notably between 2015 and 2019, followed by a modest decline in 2020–2025, while remaining above levels observed in earlier periods. A similar decline was observed in the total number of studies included in this review during the same period, suggesting that the reduction in studies employing three or more daily assessments may reflect broader publication trends rather than a decreased emphasis on repeated cortisol sampling. The overall trend toward repeated sampling use appears to be consistent with the adoption of protocols designed to capture diurnal cortisol dynamics, such as the cortisol awakening response (CAR), diurnal slope, and peak timing, which require multiple assessments across the day to adequately characterize within-day variation [38,69,70].

Despite ongoing methodological advances, considerable heterogeneity remains in the frequency of cortisol sampling in stress research [38,71]. Such variability in sampling frequency may complicate comparisons across studies and contribute to inconsistencies in the interpretation of findings [71]. Future research should therefore consider how cortisol sampling protocols can be standardized or better aligned to support more reliable comparisons across studies.

The present study has several limitations. First, the number of studies included in the analysis was relatively small, totalling 57. Although a 20-year time frame was selected to capture research trends following the increasing adoption of biological approaches to stress measurement, daily stress in typically developing children has not been a central focus of stress research, which may be associated with a limited evidence base. Therefore, findings should be interpreted with caution. In particular, the analyses were based on descriptive frequencies and percentages rather than inferential statistical testing; therefore, small differences between categories and apparent changes over time should not be interpreted as statistically established trends. Nevertheless, there is no established minimum number of studies required for trend analyses, and reporting the paucity of relevant research can itself constitute a meaningful contribution [72].

Second, a large proportion of the included studies were conducted in North America, particularly the United States. This pattern is likely influenced, at least in part, by the restriction to English-language publications, which may have introduced language bias and contributed to the overrepresentation of English-speaking regions in the sample. As a result, the observed geographic distribution does not fully reflect the global landscape of research in this area. Given evidence that stress responses and developmental outcomes vary across cultural contexts [73], future reviews should incorporate non-English literature where possible and examine cross-cultural differences more explicitly.

Third, this review focused exclusively on preschool- and elementary school-aged children (3–12 years), excluding infants and adolescents. Although this decision was made to reduce developmental heterogeneity in stress physiology, it limits the generalizability of the findings to other developmental periods.

Finally, the search and screening process may have introduced selection bias. The database search was conducted in PubMed, EBSCO Education Source, and PsycArticles to cover the main disciplinary domains relevant to this review, including medicine, education, and psychology. However, broader multidisciplinary databases such as Web of Science, Scopus, EMBASE, and CINAHL were not searched, and relevant studies indexed exclusively in these sources may have been missed. In addition, title and abstract screening was conducted by a single reviewer rather than independently by two reviewers, which may have increased the risk of inadvertent exclusion of relevant studies. Although eligibility criteria were predefined collaboratively by two reviewers and uncertain records were advanced to full-text review, this remains a limitation. To mitigate this, full-text screening was conducted independently by two reviewers and disagreements were resolved through discussion.

Despite these limitations, the present study makes several important contributions. It provides a structured mapping of methodological approaches used to assess daily stress in typically developing children over the past two decades, offering an integrated overview of how biological measures have been applied across chronic and acute stress research. By systematically synthesizing evidence across psychology, education, and medical disciplines, the review clarifies how research practices vary by stress type, age group, and biological indicator, and identifies areas where methodological approaches remain uneven or underdeveloped. Overall, the findings delineate the current landscape of biological stress assessment in children and make visible key structural gaps that characterize this emerging field.

## Conclusion

This scoping review demonstrates that biological assessment of daily stress in typically developing children has generally increased over the past two decades, with research largely centered on acute stress paradigms and cortisol-based measures. However, gaps remain in the study of chronic daily stress, the development of age-appropriate acute stress paradigms, and the use of diverse biological indicators. Addressing these gaps through methodological standardization and broader multidimensional approaches may strengthen understanding of how everyday stress becomes biologically embedded during childhood.

## Supporting information

S1 Table Original and supplementary search strategies for each database. This table provides the complete original and supplementary search strategies used in PubMed, EBSCO Education Source, and PsycArticles, including search type, search date, search terms, limits or filters, records retrieved, and notes.
(DOCX)

S2 Table Characteristics of the included studies. This table provides bibliographic information and extracted study characteristics, including year, authors, title, journal, DOI, country, type of daily stress, participant age group, data collection method, biological indicator of stress, number of daily cortisol samples, and stress measurement context.
(XLSX)

S3 Checklist PRISMA-ScR checklist. Completed PRISMA extension for Scoping Reviews checklist, indicating where each item is addressed in the manuscript.
(DOCX)
