# Supplementary material for: Methodological trends in biological assessment of children’s daily stress: A scoping review
Source: PLoS One. 2026 Aug 10;21(8):e0355460. doi: 10.1371/journal.pone.0355460 (PMC13456278; doi:10.1371/journal.pone.0355460)
Supplement: Supplementary file 1 [file pone.0355460.s001.docx]

**S1 Table. Original and supplementary search Strategies for Each Database (Title/Abstract Search)**

**Note.** This file contains database search strategies, search dates, search terms, filters, and record counts used for the scoping review. It does not contain individual participant-level data or personally identifiable information.

| Database | Search type | Search date | Search strategy / Keywords | Limits / Filters | Records retrieved | Notes |
| --- | --- | --- | --- | --- | --- | --- |
| Pubmed | Original search | 16 December 2025 | (Child*[Title/Abstract]) AND (stress[Title/Abstract]) AND (measur*[Title/Abstract] OR assess*[Title/Abstract] OR test[Title/Abstract] OR tool[Title/Abstract] OR evaluat*[Title/Abstract] OR screening[Title/Abstract] OR survey[Title/Abstract] OR questionnaire[Title/Abstract]) NOT(autism[Title/Abstract] OR asd[Title/Abstract] OR autistic[Title/Abstract] OR cancer[Title/Abstract] OR diabetes[Title/Abstract] OR traumatic[Title/Abstract] OR disorder[Title/Abstract] OR disorders[Title/Abstract] OR disability[Title/Abstract] OR adhd[Title/Abstract] OR thalassemia[Title/Abstract] OR disabilities[Title/Abstract] OR surgery[Title/Abstract] OR disabled[Title/Abstract] OR disease[Title/Abstract] OR “low-income”[Title/Abstract] OR syndrome[Title/Abstract] OR pain[Title/Abstract] OR trauma[Title/Abstract] OR HIV[Title/Abstract] OR alcohol[Title/Abstract] OR ICU[Title/Abstract] OR NICU[Title/Abstract] OR picu[Title/Abstract] OR poverty[Title/Abstract] OR disease[Title/Abstract] OR palsy[Title/Abstract] OR violence[Title/Abstract] OR infant[Title/Abstract] OR illness[Title/Abstract] OR obesity[Title/Abstract] OR overweight[Title/Abstract] OR asthma[Title/Abstract] OR malaria[Title/Abstract] OR atopic[Title/Abstract] OR dyslexia[Title/Abstract] OR abuse[Title/Abstract] OR leukemia[Title/Abstract] OR thyroiditis[Title/Abstract] OR pregnan*[Title/Abstract] OR hospital*[Title/Abstract] OR burn*[Title/Abstract] OR postpartum[Title/Abstract] OR patien*[Title/Abstract] OR “hearing loss”[Title/Abstract]) | English, 2005.1.1.–2025.12.15., classical article, observation study, humans, preschool child: 2-5 years, child: 6-12 years, full text, title/abstract search | n = 303 | Original search used for the initial review. |
| Pubmed | Supplementary poverty-related search | 20 May 2026 | (Child*[Title/Abstract]) AND (stress[Title/Abstract]) AND (measur*[Title/Abstract] OR assess*[Title/Abstract] OR test[Title/Abstract] OR tool[Title/Abstract] OR evaluat*[Title/Abstract] OR screening[Title/Abstract] OR survey[Title/Abstract] OR questionnaire[Title/Abstract])  AND (poverty[Title/Abstract] OR "low-income"[Title/Abstract] OR "low income"[Title/Abstract]) | English, 2005.1.1 – 2025.12.15., Clinical Trial, Comparative Study, Controlled Clinical Trial, Observational Study, Randomized Controlled Trial, Humans, Preschool Child: 2-5 years, Child: 6-12 years, Full text, title/abstract search | n = 67 | Conducted during revision to assess whether poverty-related terms previously included in the NOT block may have excluded relevant studies. |
| PsycArticle | Original search | 16 December 2025 | (child* AND stress) AND (measur* OR assess* OR test OR tool OR evaluat* OR screening OR survey OR questionnaire) NOT (autism OR asd OR autistic OR cancer OR diabetes OR traumatic OR disorder OR disorders OR disability OR adhd OR thalassemia OR disabilities OR surgery OR disabled OR disease OR "low-income" OR syndrome OR pain OR trauma OR HIV OR alcohol OR icu OR nicu OR picu OR poverty OR disease OR palsy OR violence OR infant OR illness OR obesity OR overweight OR asthma OR malaria OR atopic OR dyslexia OR abuse OR leukemia OR thyroiditis OR pregnan* OR hospital* OR burn* OR postpartum OR patien* OR "hearing loss") | English, 2005.1.1.–2025.12.15., journal article, humans, childhood, preschool age, school age, full text, title/abstract search | n=147 | Original search used for the initial review. |
| PsycArticle | Supplementary poverty-related search | 20 May 2026 | (child* AND stress)  AND (measur* OR assess* OR test OR tool OR evaluat* OR screening OR survey OR questionnaire)  AND (poverty OR "low-income" OR "low income") | English, 2005.–2025., academic journal article, article, peer reviewed journals, full text, human, preschool age (2-5 yrs), school age (6-12 yrs), title/abstract search | n=5 | Conducted during revision. |
| EBSCO education | Original search | 16 December 2025 | (child* AND stress) AND (measur* OR assess* OR test OR tool OR evaluat* OR screening OR survey OR questionnaire) NOT(autism OR asd OR autistic OR cancer OR diabetes OR traumatic OR disorder OR disorders OR disability OR adhd OR thalassemia OR disabilities OR surgery OR disabled OR disease OR "low-income" OR syndrome OR pain OR trauma OR HIV OR alcohol OR icu OR nicu OR picu OR poverty OR disease OR palsy OR violence OR infant OR illness OR obesity OR overweight OR asthma OR malaria OR atopic OR dyslexia OR abuse OR leukemia OR thyroiditis OR pregnan* OR hospital* OR burn* OR postpartum OR patien* OR "hearing loss") | English, 2005.1.1.–2025.12.15., academic journal article, article, full text, title/abstract search | n=855 | Original search used for the initial review. |
| EBSCO education | Supplementary poverty-related search | 20 May 2026 | (child* AND stress)  AND (measur* OR assess* OR test OR tool OR evaluat* OR screening OR survey OR questionnaire)  AND (poverty OR "low-income" OR "low income") | English, 2005.1.1.–2025.12.15., academic journal article, article, peer reviewed journal, full text, title/abstract search | n=225 | Conducted during revision. |
